# Public attitudes towards pricing policies to change health-related behaviours: a UK focus group study

**DOI:** 10.1093/eurpub/ckv077

**Published:** 2015-05-16

**Authors:** Claire Somerville, Theresa M. Marteau, Ann Louise Kinmonth, Simon Cohn

**Affiliations:** 1 Institut de Hautes Etudes Internationales et du Développement, Global Health Programme and Programme for Gender and Global Change, Graduate Institute of International and Development Studies, Geneva, Switzerland; 2 Behaviour and Health Research Unit, Institute of Public Health, University of Cambridge, Cambridge, UK; 3 Health Services Research and Policy, London School of Hygiene and Tropical Medicine, London, UK

## Abstract

**Background**: Evidence supports the use of pricing interventions in achieving healthier behaviour at population level. The public acceptability of this strategy continues to be debated throughout Europe, Australasia and USA. We examined public attitudes towards, and beliefs about the acceptability of pricing policies to change health-related behaviours in the UK. The study explores what underlies ideas of acceptability, and in particular those values and beliefs that potentially compete with the evidence presented by policy-makers. **Methods**: Twelve focus group discussions were held in the London area using a common protocol with visual and textual stimuli. Over 300 000 words of verbatim transcript were inductively coded and analyzed, and themes extracted using a constant comparative method. **Results**: Attitudes towards pricing policies to change three behaviours (smoking, and excessive consumption of alcohol and food) to improve health outcomes, were unfavourable and acceptability was low. Three sets of beliefs appeared to underpin these attitudes: (i) pricing makes no difference to behaviour; (ii) government raises prices to generate income, not to achieve healthier behaviour and (iii) government is not trustworthy. These beliefs were evident in discussions of all types of health-related behaviour. **Conclusions**: The low acceptability of pricing interventions to achieve healthier behaviours in populations was linked among these responders to a set of beliefs indicating low trust in government. Acceptability might be increased if evidence regarding effectiveness came from trusted sources seen as independent of government and was supported by public involvement and hypothecated taxation.

## Introduction

Evidence from systematic reviews, meta-analysis and time-series studies consistently supports the use of pricing interventions in achieving healthier consumption patterns among the population in relation to alcohol in excess, cigarettes and, to a lesser extent, for food.^1–8^ Interventions are most often implemented by government through additional taxation on items where consumption contributes to risk of non-communicable disease (NCDs). This policy is endorsed by the World Health Organization (WHO).^9–13^ In this article, pricing policy consequently refers to the use of taxation to influence health-related behaviour.

Evidence of negative public attitudes towards such policy interventions is accumulating across Europe and elsewhere.^14–20^ An in-depth understanding of these attitudes is necessary to inform effective implementation. The aim of this study is consequently to draw on qualitative data to explore the nature of public acceptability, and in particular those values and beliefs that potentially compete with evidence presented by policy-makers. Gaining a sense of people’s perspectives is of central importance because people are never neutral or passive recipients of policy, but are likely to reinterpret and respond to such initiatives in diverse and subtle ways.

## Methods

We chose a focus group (FG) method appropriate for examining attitudes and beliefs about health,^18–22^ by which differences of opinion and debate are likely to be triggered by the set of topics under discussion.^21–24^ Participants were recruited by a research agency from inner and outer London. In order to gain as wide a range of views as possible each group was heterogeneous according to such things as ethnicity and gender, but, following a standard FG approach, was constituted by something in common; we chose income (as a proxy for SES), using The Market Research Society’s occupational categories.^25^ Given the sampling strategy, mean average income is not intended to be representative of the population. Participants completed a short self-report demographic questionnaire and were reimbursed for their time, travel and childcare costs.

**Figure 1:**
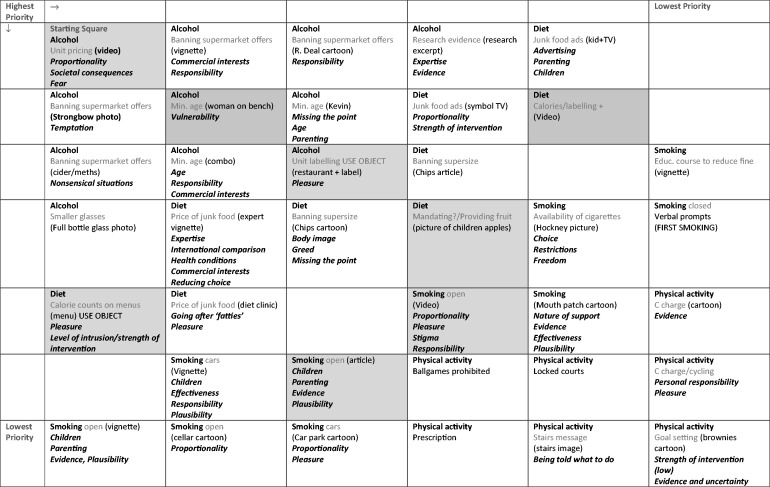
Discussion guide for focus group

Sessions began with a standard introduction to the topic, followed by a warm-up exercise and then visual and textual stimuli to provide a set context for the expression and discussion of diverse views.^26^ Groups followed the same protocol, although specific prompts varied according to a topic ‘road map’ (figure 1). Real and fictional stimuli drawn from a systematic review^27^ included: a short video; a selection of photographs and other images relating to alcohol in excess, cigarettes and food; and text extracts from media and research sources (see website for materials). The experienced facilitators encourged conversation and debate with minimal intervention, other than to introduce new topics or seek clarification.

Participants gave written consent for discussions to be video and audio recorded, and study details to raise concerns subsequently. Because this research was not conducted with NHS patients, it fell outside the national UK research ethics committee process. Instead, it fell under institutional governenance and was approved by the Cambridge Psychology Research Ethics Committee, Council of the School of Biological Sciences, University of Cambridge (7/11). Participants chose their identity or were allocated pseudonyms.

## Analysis

Audiotapes were transcribed verbatim by an independent service. Analysis was conducted by two experienced data analysts, a principal investigator on the study who co-ran the focus groups (SC) and an independent analyst who was not present at the focus groups (CS, lead analyst) who had access only to the raw transcripts. CS read all transcripts for data immersion and held eight documented ‘hashing-out’ discussions with SC.^28^ These discussions refined the constant comparative approach to data sorting and resulted in definitions for first order themes. CS then systematically applied these themes to three randomly selected transcripts to generate subcategories under each theme. Analysis continued as transcripts were read, with team discussions generating detailed coding to understand how themes related. Data were extracted, sorted and categorised and saved in files on a shared server to enable remote monitoring. A shared data analysis diary was maintained and used to enable a constant comparative process so emergent themes could be interrogated.

## Results

Twelve focus groups, each consisted of 7–10 participants, were conducted between October 2011 and February 2012. Ninety-four participants (48 men, 46 women) aged 19–68 years were recruited in total, with self-reported household incomes ranging from £8000 to £250 000 per annum. The majority of participatns were healthy and reported that they had consumed some alcohol during the previous month; 27 were current smokers (table 1).

**Table 1:** Demographic characteristics of the focus group participants

Total	94
Age range	19–68 years (mean 39.5 years)
Sex	Male 48, female 46
Nationality	British: 88; Nigerian: 1; Jamaican: 1; Sierra Leonean: 1. (3 missing)
Current employment	Full-time: 48, part-time: 10; self-employed: 4; student: 2; unemployed: 14; retired: 6. (10 missing)
Ethnicity	White: 53; Back/Black Caribbean: 17; Mixed: 4; Asian: 3, (17 missing data)
Household income range	£8000–£250 000; (mean £52 000 of 68 respondents)
Main source of income	Salary: 57; government benefits: 12; pension: 9, savings: 2 (14 missing data)
Home status	Owner occupier: 53; renters: 29; Living with parents: 5 (7 missing)
Alcohol consumption	Drinker: 70 (ranging from ‘everyday’, ‘about 15 units a week’ to ‘weekends only’ or ‘about a bottle of wine a month’); non-drinker: 21 (3 missing)

In addition, 12 lived alone, 27 were current smokers and 11 described themselves as having an on-going illness of some sort (such as asthma, arthritis and back pain)

Acceptability of pricing policies was most explicitly addressed in three themes: *Relationship to Government* (descriptions that personify or narrate the relationship of ‘us’ vs. ‘them’); *Collective Suffering* (descriptions referring to a collective sense of lack of well-being or suffering as a possible explanation for unhealthy behaviour); and, *Taxation and Pricing* (descriptions that depict an unhelpful or problematic relationship between linking health, price and taxation). Together 22 000 words, 12% of total transcribed utturences, were devoted to these three themes. Analysis of the interactions and links made between these themes confirmed an overall unfavourable attitude and low level of acceptability to the use of fiscal policies to change behaviour.

Participants elaborated on three sets of beliefs associated with these expressed negative attitudes:

- Pricing makes no difference to behaviour.
- Government operates as an enterprise and introduces pricing policies to generate income (not changes behaviour)
- Government and the evidence it cites is not trustworthy.

These beliefs appeared to guide responses to policy proposals more strongly than notions of individual or population health. A sceptical remark about a policy proposal typically led to shared expressions questioning government motivation. This underlying stance was shared by virtually all the participants across themes and in relation to policies about all behaviours.

### Belief that pricing will make no difference to behaviour

Most respondents suggested that increases in the price of unhealthy products whether it was alcohol, cigarettes or high fat and unhealthy food, would have no impact on consumption. Reasons expressed included the addictive nature of the products with arguments that people were likely to sacrifice other expenditures in order to maintain their consumption or find alternative, cheaper ways to procure them. Typical phrase include; ‘*They’ll never stop people doing what they want to do. (FGD064)’* and *‘It’s not going to stop people (FGD066)’.*

Although there was not always consensus on the incapacity for pricing to change consumption patterns, when a participant tried to talk through if such a strategy might work there always followed a lively debate, with others challenging this viewpoint. Participants were thus fairly entrenched in the position that price has no impact on behaviour (see Box 2) .

### Belief that the government operates as an enterprise: ‘Government Inc.’

The claim that pricing would not be an effective way to change behaviour was also understood in direct relation to the idea that taxation is always primarily about revenue or ‘profit’ (see Boxes 1 and 2). There were some positive allusions to hypothecation of taxes to improve relevant health services (see Nigel in Box 1). Suggestion that pricing policies were designed to address health issues was rapidly dismissed as merely a means to justify and legitimate the underlying government motive. As one participant summarized: *‘I think as well, the government pretend they care but really it’s their job to, they don’t really care, they just care about earning money … ’ (FGD061). Such discussions demonstrate the interlinking of themes (1) and (2): pricing makes no difference to behaviour and government operates as an enterprise that introduces pricing policies to generate income (see **Box 1**).*

**Box 1:** Discussions on smoking and food consumption behaviour demonstrating the interlinking of themes; (1) and (2); pricing makes no difference to behaviour and government operates as an enterprise and introduces pricing policies to generates income

**Sandra***: It’s like smoking, they put the tax on, it’s not going to stop anyone.*
**Paul***: Jacking it up and jacking it up and jacking it up, they won’t stop, you know.*
**Sandra***: No.*
**Nigel***: But the price is not a deterrent, but I think I sort of took it as being a tax to pay for the extra … raise extra revenues for the health service, that’s what I took it as, you’re saying a fat tax, I thought they were trying to … maybe … maybe I’m wrong.*
***Patrick****: Well, if it’s … if it’s …*
**Graham***: I don’t think it’s going to stop people from eating the food.*
**Nigel***: It’s certainly not a deterrent, 8p or something.*
*(FGD073)*	
**Aiden:** *Smoking, yeah, there’s just too much money they’re making out of it to stop it.*
**Mary**: *And they don’t want to lose out on that money.*
**Aiden: ***You know if you’re worried about it that much, stop selling fags but they won’t do that because their making so much money, it’s bringing, do you know what I mean.*
**Jock:** *Yeah and not only that, think about the knock on effect … . They don’t really want people to give up smoking so it’s contradictory isn’t*
**Aiden:** *So what are you saying that smoking is like the feeding arm of the NHS in a way?*
**Jock:** *I am, yeah.*
**Aiden:** *That’s a better way of looking at things is it?*
**Jock:** *No, but I’m not, unfortunately the truth of the matter is that is the realistic thing, the fact that they want you to give up smoking and if you look at the amount they’re spending to stop people smoking it’s peanuts, absolute peanuts because they’re making billions, they’re not making millions, they’re making billions so they spend two million to say “hey look at us, we’re trying to stop smoking”. No you’re not, you don’t want to stop it. (FGD067)*

**Box 2:** Dialogue that illustrates beliefs around distrust in government and contradictions in goverment policy around alcohol

**Thea:** *No, carry on.*
**Bradley:** *More and more people seem to be going out late, getting drunk indoors and then going out with say thirty pounds and using that as a cab home, you know, rather than go out drinking, staying in is a lot cheaper because they keep putting the prices up, putting the prices up and people just get sick of paying what they want.*
**Fac 1:** Yeah.
**Thea:** *This is the, um, same government who don’t want us to binge drink but have extended the licensing hours in all the pubs. So, people don’t have to go out until ten, eleven at night because all the pubs are now open until two, three … Well, not really where I live but if you go into towns, they’re open till two, three, some of them four o’clock in the morning so it’s a bit of a … It’s a bit of a hypocritical thing for them to say.*
**Omer:** *I don’t think it’s a problem with the long hours, is I agree with the gentleman who says is it’s to do with the education, to educate people how to drink because if you go all over the Europe, Italy, France, people they drink there with common sense, you know, they have a glass of wine with their meal and then they go in the bars and stuff, they have a glass, they have a laugh and I used to work as a bouncer and I know how it is because obviously you got … You got people bringing drinks in their bags, to go into the club because it’s expensive and stuff like that. But it’s to do with education, to do … To tell the people that you can’t just drink because I was talking to people and saying … I was asking, why do you want to get drunk? Why? Because it’s a … It’s a fashion, it’s kind of if you’re not getting drunk with the mates you’re kind of weak so that kind of stuff is kind of trend to get drunk and get smashed *…
**Fac 1:** *Does anyone else agree with that view about education or disagree?*
**Soji:** *Yeah, sure … Yeah, government should do more than just increasing the price, educating people, the effects of alcohol, um …*
**Omer**: *Because we go back to what happened in America in the forties, fifties, whatever was that time that they restricted the alcohol and stuff like that. If government does put the price up you can always have in a black market cheap stuff and people will always get that. So like I said, there has to be …*
**Georgia:** *Culture change.*
**Omer:** *Culture change. Education and then tell people how to drink, don’t make a drink as an evil thing but reasonable to …*
*To be honest with you I don’t trust government whatsoever because obviously whoever comes into the …*
*(Laughter)*	
**Omer:** *Into the party, they just do their own stuff. And whatever you say to government, one person does something today, next government comes in four years’ time, five years’ time, they change things. So obviously whatever we say that doesn’t count, they do their own stuff. People like us … People like us, they don’t listen. So we have those debates and stuff like that, by the end of the day they talk to themselves and they … Probably they make a decision, those decisions are made from people who probably they’re not asked to see those things. Like I said, I’ve been … I was working in those places for seven, eight years and I’ve seen how people drink and how drink affects people and, and, and, and even at clubs and stuff like that, no matter what prices you put, high or lower. You got a student now, it’s two pound for, for vodka and a Red Bull, for students …*
(FGD 061)	

Discussions of taxation being about revenue rather than health protection through behaviour change sometimes led to proposals that prohibition was the only way to change behaviour, but might only be acceptable where behaviours were presented as addictive, harmful drugs like smoking. While prohibition was seen to helpfully prioritise health protection over taxes, there was a counter-argument expressed about government as a detached entity that controls and tries to determine everyday social behaviour as either healthy or unhealthy. The language here invoked feelings of subjugation and loss of agency. Sonia describes this as *‘the government hypnotising us*’ and Gina in the same dialogue as *‘giving up things, that’s what it always remind me of, government kind of policies is all about giving up smoking, giving up this, giving up that’* followed by Maria as ‘*general restrictions, it just feels like you’re more and more restricted for the things you want to do for enjoyment, when they enforce all the policies’* (FGD 061). Brian (FGD 071) feels that there is ‘*government interferes in every facet of your life, even those that are pleasurable’* .

Despite such arguments against the idea of a ‘nanny state’ some felt the cost to the state, and more specifically the limited resources of the National Health Service (NHS), justified the banning of some products but acknowledged difficulties with alcohol.

**Table:** 

**Anna:**	*like smoking it’s just going to give you, you know, make you ill but the thing is that I think it’s very difficult to stop people drinking, I don’t know how you would, you know, you’d end up in a nanny state where you know. And if you start telling bars they can’t serve people, can you imagine the ructions there’s going to be?*
**Fac 2**:	*So that does mean we should ban smoking altogether?*
**Cate**:	*Absolutely. (FG067)*

An argument was repeatedly made that the government would shy away from prohibition to protect revenue, particularly for the health service. The idea that either the government or, at the very least the National Health Service, would ‘*go bang’* or ‘*flat line’* (Jock FGD 067) or become ‘*skint’* (Jimmy FGD 070) if additional taxes on unhealthy products were to reduce consumption supported the belief that the purpose of price-related policies that reduced consumption was only to ‘*make money’* (Sid FGD 072). This in turn gave rise to the view that the government operates in ways that resemble big businesses that supply unhealthy products ‘*just as MacDonalds’*. The view that in some way government is profiting from the suffering masses was expressed in several discussions across the focus groups. For example:Any suggestion that the government operated in ways to protect the public for example by making healthier foods cheaper, was notably absent from the discussions.

**Table:** 

**Bradley:**	*It’s on the government’s conscience because they’re the ones that are selling the product that’s killing so many thousands of people per year. Or maybe millions, I don’t know the statistics but they’re selling this stuff, which is deadly, which will eventually kill people and they’re earning money off of it (FGD 062).*

**Table:** 

**Vic***:*	*I think it’s lip service because they’re just about making the money because they’ll make the money more that way* [putting up taxes] *than making healthy food cheaper. So it’s the government being the government.*

Sometimes suspicion was developed further, suggesting that the government sought to extract income by stealth; as Natalie contends, ‘*Every time the government do something I just think they’re doing it for their own benefit, they just want your money … That’s all they want is that extra money (FGD 068).* Similarly*,* in closing one discussion Thea sums up the group’s collective dismay at the ‘*hypocrisy’* of a government that extends licensing hours whilst wanting to reduce binge drinking; bans tobacco advertising but not alcohol promotion; sets guidelines on healthy alcohol consumption but not cigarette consumption [paraphrased from Thea, original in Box 2].

Every time the budget comes out, along with alcohol and fuel, everything goes up in price … But that just goes straight back into the government’s pockets so, to be spent on God knows what … if it wasn’t smoking it would be something else so, food, fuel, you know, utilities are just … (Tina FGD 061.)

### Belief that the government is not trustworthy

The data suggest strongly that the government simply cannot be trusted:This lack of trust is exacerbated by the perceived contradiction between policy and motive when it comes to matters relating to health:The lack of trust extends to be a general underlying logic by which participants interpret virtually every issue raised. For example, in response to a brief summary of systematically reviewed evidence of alcohol pricing and consumption, concluding that minimum price per unit of alcohol would result in significant reductions in consumption, none of the participants in the 12 focus groups believed the statement and expressed distrust in relation to any data or research deemed to be sourced from government. The argument related not to the quality of evidence but to its contradictory interpretation and the selective sourcing by government of evidence to suit its purpose (See Box 3).

**Box 3:** Dialogue that illustrates distrust in governance including doctors

**Mike:**	*… And that’s how it is, you know, divide and conquer … Look, use an independent party that’s non-government related and make it obvious about that. You might try … We might start trusting them a little bit but with the government’s hand in it. (Intake of breath) I don’t think we want … It’s just sticky fingers everywhere. Fingerprints all over it.*
**Mic:**	*You know, what I was going to say is that, you pick up a newspaper and you’ll read that there’s been research done by, let’s say, the BMA [the British Medical Association], they come out with these certain findings. Few days or a few weeks later, you find another group of doctors from another organisation will come out with totally the opposite view.*
**Vic:**	*There’s always contradictions.*
**Mic:**	*Where are you led to believe? Which is right, which is wrong? In other words, what happens in most cases, everyone just carries on as they have been doing.*
	*(Laughter)*
**Craig:**	*But why would you believe? As you say, it’s always contradictory.*
**Mike**:	*I read the other day that water was bad for you. Imagine that. Water’s bad for you.*
**Vic:**	*See, there you go.*
*(Laughter) (FGD066)*

**Table:** 

**Olan:**	*To be honest with you I don’t trust government whatsoever (FGD 061)*
**David:**	*I’d rather trust myself than the government. (FGD 061)*

**Table:** 

**Gina:**	* … , if you say, you know, on the one hand they’re saying, don’t smoke in public places, on the other hand they’re still allowing fags to be sold in shops. They’re really giving a contradictory message, aren’t they? I mean, what is it they’re actually trying to say? (FGD 061)*

The belief that government cannot be trusted, is consistent with the previous two beliefs identified and was held across groups. Any dissenting views expressed, any potentially contrary evidence or information, or further probing by one of the facilitators was simply reframed and reinterpreted to uphold the belief. Thus, perhaps more so than the previous two, this belief seemed immutable.

## Discussion

These focus groups confirmed evidence from across Europe and elsewhere of the low acceptability of pricing interventions aimed at changing health-related behaviour.^27^ We demonstrate an association of this negative attitude with three consistently expressed and interconnected sets of beliefs: that pricing policies will make no difference to behaviour; that government prioritises its fiscal responsibility over responsibility for the welfare of its constituents; and that government is untrustworthy. The word ‘belief’ is used here to define dispositions rather than informed positions.^28^^,^^29^ In the focus groups—like any other social context—beliefs take on a social form, being established and expressed through interaction with others, and based on emotional and symbolic engagement as much as a rational, information-based assessment. Demonstrating the importance of such cultural factors therefore complements other approaches to alcohol policy that forground such things as the lack of policy infrastructures or public lack of knowledge about the health effects of alcohol.^30^^,^^31^

Beliefs expressed were largely consistent across behaviours. Participants talked about pricing policies as ineffective at changing health-related behaviour but as highly effective ways to increase government income. The lack of trust appeared to emanate from a perceived inconsistency between the role of government as a collector of tax and as the steward of the population’s wellbeing. The three beliefs were marshaled in various combinations to articulate suspicion in relation to this core tension. The characterisation of government as a single homogeneous body, with common goals and single motives, enabled participants to establish a simple narrative that, during discussion with others, served to consolidate a generalised view. Although these beliefs can be interpreted as justifying, and possibly obfuscating, people’s reluctance to pay more for behaviours they enjoy by eschewing the claim such policies might benefit health, the strength of distrust for government should not be underestimated. For example, only four of the 94 participants ever expressed any response classified as favourable to government, and no group ever came to a consensual view in favour of policy makers.

Our data support the view that responses to policies are shaped by social context and a broader set of judgments, rather than simply that normally defined as evidence.^32^ Indeed, as also reported by others, the presentation of evidence suggesting the effectiveness of fiscal policies to reduce unhealthy behaviours only stimulated discussion about its potential for contradictory interpretation and the selective sourcing of evidence by government to suit its purpose.^33^

### Study strengths and limitations

The focus group design effectively captured group responses to diverse stimuli related to policy to reduce the consumption of alcohol, cigarettes and unhealthy foods. The method enabled participants to discuss, debate, reflect and exchange a range of positions and experiences. Data thus consist of the ways in which topics were engaged in through interaction, and the manner in which utterances were picked up and built upon by others. This allowed identification of underlying beliefs and their interrelationships that would not be captured by other methods. As a result, a core strength is that it problematises the idea that public acceptability is ever simply determined by the strength and nature of supporting evidence, and instead illustrates the extent to which it emerges as an inherently cultural and social assessment based on a wide heterogeneity of factors.

Use of a data analyst who was blinded to the original research proposal, sampling and data collection (CS), alongside a more engaged researcher (SC) contributed to the inter-rater reliability and rigor of the analysis. Participants were selected according to gender, ethnicity and SES in order to capture a sense of the metropolitan population and thus ensure as wide a variety of potential views were included as possible: The sample was relatively large for a qualitative study of this kind. Caution is necessary however, both in terms of the interpretation of the beliefs identified and in the generalisation of the findings to other populations and behavioural targets of government fiscal policies.

### Research and policy implications

Our findings demonstrate the complementary role of qualitative to quantitative methods in policy acceptability studies. Further research could draw on these findings to consider how beliefs are socially produced by, and may shape responses to, fiscal policies and the evidence for and against them, in the sphere of behaviour change and public health.

A range of research suggests the public are more accepting of fiscal policies the larger their effect on health and health-related behaviours and that this is true of financial incentives as well as taxation^34^ This study illustrates how acceptability can also relate to underlying beliefs about the policy maker, in this case government. Mistrust can extend from the evidence of effectiveness itself to the uses of the revenues raised. Acceptability is associated with health hypothecation of taxes^35^ more work is needed to test the relationship experimentally.

Policy acceptability might best be conceived as a broad disposition that emerges and is sustained from diverse factors other than the aims of policy or the presentation of associated evidence alone. Research which clarifies the complex relationship between beliefs, public acceptability and government action is likely to span historical and sociological as well as psychological perspectives in order to unravel the power of a wider set of factors, including industry, advertising and the media.

Meanwhile this study adds voice to a range of possible ways to work with the public on acceptability of pricing policies in addition to a focus on health gain. These include greater transparency of government both in use of the revenues by hypothecation of taxes for societal purposes and in pre-policy planning and preparation.
